# A novel high-power all-fiberized flexible spectral filter for high power linearly-polarized Raman fiber laser

**DOI:** 10.1038/s41598-018-28686-2

**Published:** 2018-07-19

**Authors:** Jiaxin Song, Haiyang Xu, Jun Ye, Hanshuo Wu, Hanwei Zhang, Jiangming Xu, Pu Zhou

**Affiliations:** 1 College of Advanced Interdisciplinary Studies, National University of Defense Technology, Changsha, 410073 China; 2 Hunan Provincial Collaborative Innovation Center of High Power Fiber Laser, Changsha, 410073 China

## Abstract

Power scaling of linearly polarized Raman fiber laser (LPRFL), which has wide application potentials, is mainly limited by the generation of high-order Stokes light. In this paper, we propose a novel flexible spectral filter with all-fiberized configuration and high-power handling. Combining with the polarization-dependence of Raman gain, the filter could be used to efficiently suppress high-order Stokes light in LPRFL and thus help further power scaling. The filter is fabricated by two 45° cross-splice of three pieces of polarization maintaining (PM) passive fibers. The bandwidth and central wavelength of transmission spectrum of the spectral filter could be flexibly tuned through changing the length and temperature of the cross-spliced fiber. The insertion loss of the filter fabricated in the lab is measured to be as low as 0.07 dB. The filter is employed in a LPRFL, and the maximum output power of the LPRFL is increased by 48.7%.

## Introduction

High power Raman fiber lasers have attracted much attentions in recent years, and have wide applications in optical communication, supercontinuum generation, special wavelength source and medicine^1–3^. At present, the output power of pure-Raman fiber laser is up to several hundred watts^3–7^. However, the maximum output powers of linearly polarized Raman fiber laser are usually lower than their randomly polarized counterparts^8^. Nevertheless, linearly polarized Raman fiber lasers (LPRFLs) are required in many fields such as frequency conversion, spectroscopy, spectral beam combining, coherent detection, communications, and so on^8–10^. The output power of linearly polarized Raman fiber laser is relatively low^10–12^. S. A. Skubchenko *et al*. demonstrated 4.7 W LPRFL centering at 1120 nm wavelength with conversion slope efficiency of 87% in 2004^11^. In 2016, A. A. Surin *et al*. reported 14 W output power at 589 nm through frequency doubling of 65 W output power of LPRFL operating at 1178 nm^10^. In addition, IRE Polus Group (IPG) Photonics Corporation has produced the Raman Laser Module (RLM) series and Raman Laser Rack (RLR) series^13^, which can deliver up to 500 W output and linearly polarized operation is optional. But they did not reveal any technical details, so it is not taken into consideration when making comparison. In addition to the complex manufacturing process and  high cost of polarization maintaining (PM) fibers and devices, relatively high Raman gain of LPRFL leads to low threshold of high-order Stokes light^14^, which becomes a main limitation for further power scaling. Using hybrid (Yb-Raman) gain Raman fiber laser is a good way to demonstrate high power scaling of Raman laser. Several kilowatts output was obtained in randomly polarized operation in Yb-Raman fiber laser^15–17^. As for linearly polarized operation, the output power has also been scaled up to kilo-watt level^18^. Since part of pump light would injected from inner cladding to the core, Yb-Raman fiber laser is not classical core-pumped Raman fiber laser. What we mainly focus on is linearly polarized core-pumped pure-Raman fiber laser, so we would not take Yb-Raman into account in this paper.

At present, there are mainly two approaches for suppressing high order Stokes light. One is using specially designed fiber such as W-type fiber and dual-hole-assisted fiber with large loss at long wavelength^3,19–21^. This method requires special manufacturing techniques and splicing method. Another one is employing lumped spectral filters such as long period grating and tilted fiber gratings in order to couple the high order Stokes light from core mode to cladding mode^22–25^. However, the fabrication process of the spectral filters is relatively complex. In addition, lumped filters would lead to substantial signal insertion loss while each filter element may have relatively low insertion loss such as 0.2 dB^22^. As far as we know, these two methods have not been applied in high power LPRFL yet. LPRFL generally adopts polarization maintaining structure, therefore both the filter fiber and grating require new design, which is also a difficult work to be accomplished.

In this paper, we achieve efficient suppression of the high order Stokes light in high power LPRFL based on a novel all-fiber spectral filter. The filter is realized by two 45° cross-splicing of three pieces of polarization maintaining (PM) passive fibers. Through this filter, the polarization direction of high order Stokes light could be manipulated to be orthogonal to that of its pump light, then Raman gain would be decreased^14^. By tuning the fiber length and temperature, the bandwidth and the central wavelength of the filter could be controlled flexibly. Theoretical calculation shows that this filter has the potential to suppress Stokes light effectively. The transmission spectrum of the filter is measured by injecting wideband optical source. A proof-of-concept experiment is carried out to verify the feasibility of decreasing the power of Stokes light by using this spectral filter and optimize the position of the filter in the amplifier. The spectral filter is employed to suppress the second order Stokes light (1177.0 nm), and as a result the maximum output power of the first order Stokes light (1119.6 nm) is increased by 48.7% compared with the absence of the spectral filter.

## Results

### Measurement of transmission properties of the filter

**Figure 1:**
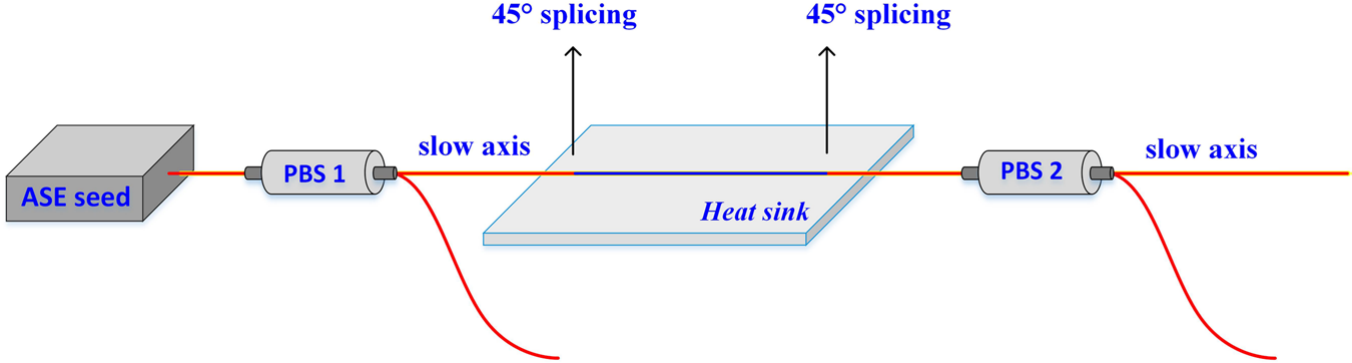
The measurement setup of transmission spectrum. ASE: amplified spontaneous emission; PBS: polarization beam splitter.

The measurement setup of transmission spectrum of the filter is depicted in Fig. 1. A home-made amplified spontaneous emission (ASE) source was used to provide wideband pump source. The light propagating along the slow axis was selected by the two polarization beam splitters (PBSs). The filter was consisted of three pieces of PM passive fiber connected by two 45° cross-splicing. A piece of 80 cm long commercial polarization maintaining passive fiber was employed to provide phase difference. The core and cladding diameters of the fiber are 10 and 125 μm, respectively. The temperature of the cross-spliced fiber is controlled by a heat sink.

**Figure 2:**
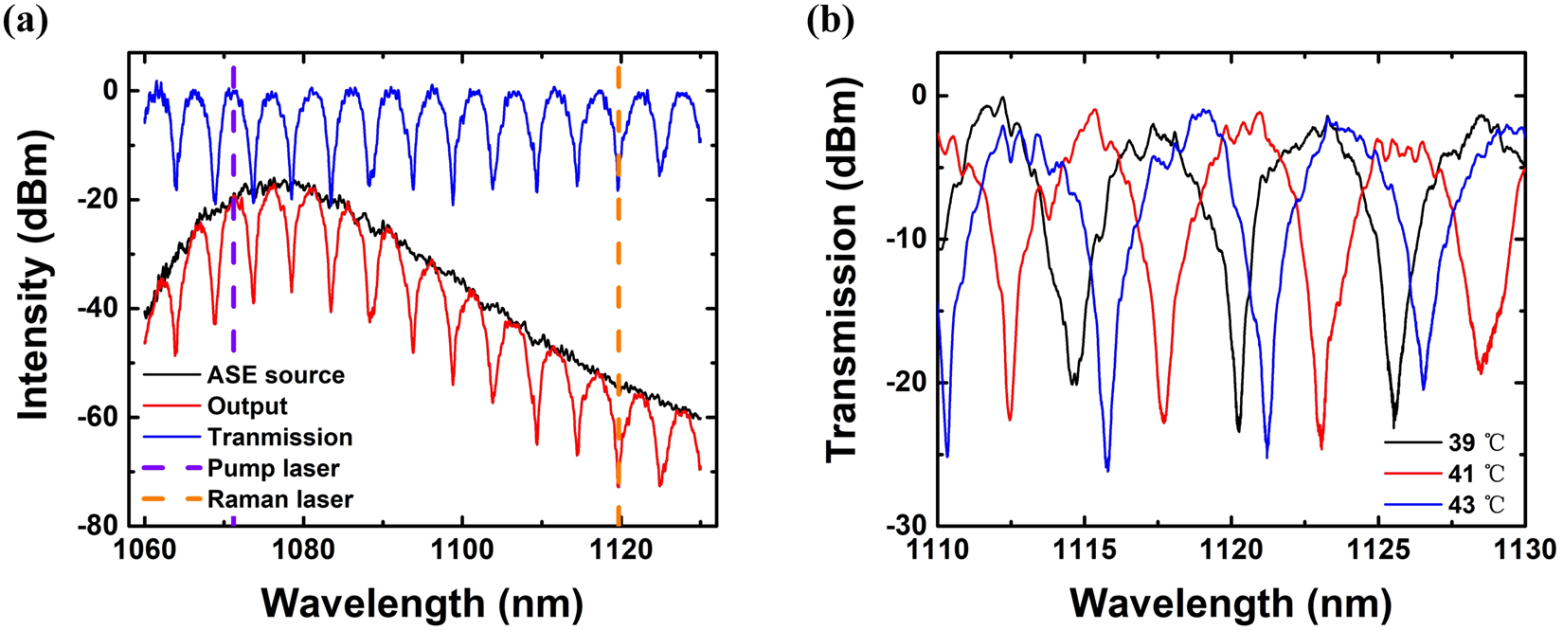
The measured transmission spectrum at (**a**) 17 °C: ASE pump source (black), output laser (red), transmission spectrum (blue), pump laser (dashed violet), Raman laser (dashed orange); (**b**) different temperatures: 39 °C (black), 41 °C (red), 43 °C (blue).

When the temperature of the heat sink is set to be 17 °C, the transmission spectrum can be obtained by calculating the difference between the output laser and input ASE source, the result of which is shown in Fig. 2(a). This transmission spectrum implies that the potential Raman suppression ratio with this filter may be as high as 20 dB. The spacing between adjacent peaks is about 5 nm. Through theoretical calculations, the appropriate length and temperature are selected to ensure that the transmittance of pump light is constant while the transmittance of Raman light is rather low. The selection of length and temperature results in low transmittance for Raman laser and high transmittance for pump laser. It means that the Raman light would mainly transfer to the fast axis, while the polarization direction of pump light would keep unchanged. The relationship between transmission spectrum and temperature was also investigated. As is shown in Fig. 2(b), the transmission spectrum shows a trend of blueshift by ~ 1 nm when the temperature increases by 1 °C. By changing the temperature, the transmittance at a certain wavelength could be tuned flexibly. The insertion loss of the filter is measured to be around 0.07 dB, which can be attributed to the splicing loss.

### Proof-of-concept experiment

**Figure 3:**
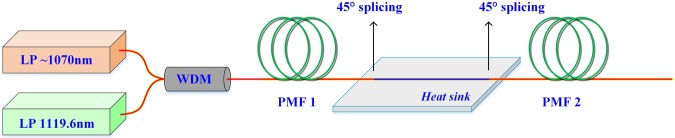
The experimental setup of Raman fiber amplifier. LP: linearly polarized; WDM: wavelength division multiplexer; PMF: polarization maintaining fiber.

In order to verify the feasibility of suppressing Stokes light by using the proposed filter, a proof-of-concept experiment was carried out, and the experimental setup is depicted in Fig. 3. The filter measured above was inserted into a piece of PMF with the total length of 400 m in a Raman fiber amplifier to verify the effect on suppression of Stokes light. The seed laser (Stokes light of the Raman fiber amplifier) is a 1119.6 nm Yb-doped fiber laser polarized along the slow axis. The output power of the seed laser is 300 mW. The pump source is a home-made linearly polarized tunable Yb-doped fiber amplifier based on master oscillator power amplifier (MOPA) structure. The pump source and seed laser are copolarized and the output end of them are injected into the corresponding input ports of a 1070/1120 nm wavelength division multiplexer (WDM). The filter is spliced between two pieces of commercial 10/125 μm (core/cladding diameter) PM passive fiber.

**Figure 4:**
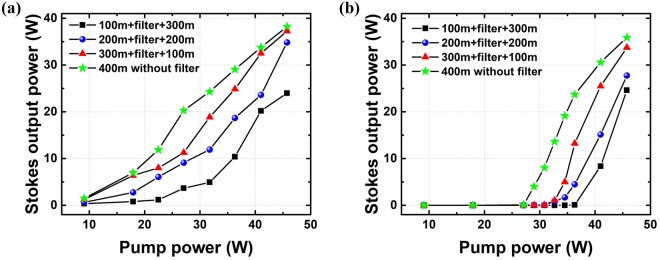
The output power of Stokes light with (**a**) 300 mW seed laser; (**b**) no seed laser at different positions.

The output power of Stokes light versus pump power in the Raman fiber amplifier is shown in Fig. 4(a). The maximum output power without a filter is 38.2 W corresponding to the conversion efficiency of 83.6%. When the length of PMF 1 is 100 m and the length of PMF 2 is 300 m, the maximum output power of Stokes light is 24.0 W corresponding to a conversion efficiency of 52.5%. The conversion efficiency decreases by 31.1% owing to the use of this filter. By keeping the total length of passive fiber unchanged, the influence of position of the filter on suppression effect of Stokes light was also explored. A conclusion can be drawn from Fig. 4(a) that the longer the length of PMF 1, the stronger the suppression effect. This relationship may be explained by the excessive deflection of the Stokes light when PMF 1 is longer. When the length of PMF 1 is longer than 100 m, too much Stokes light is deflected by the filter and the gain between the two polarization directions would work. Then the suppression effect would be weakened. Since high order Stokes light is usually generated by spontaneous Raman gain, it is necessary to study the suppression effect of the filter on Stokes light in Raman laser without a seed laser. The output power curve is depicted in Fig. 4(b). The maximum output power of Stokes light without a filter is 35.9 W and the conversion efficiency is 78.5%. However, when the length of PMF 1 is 100 m, there is merely 24.6 W Stokes light generating at the same pump power, which corresponds to the conversion efficiency of 53.8%. The relationship between the position of the filter and the suppression effect in this experiment is the same as the previous one with a seed. The above results prove the feasibility of suppressing high order Stokes light by exploiting the proposed filter in the amplifier.

### Experiment on suppressing the second order Stokes

**Figure 5:**
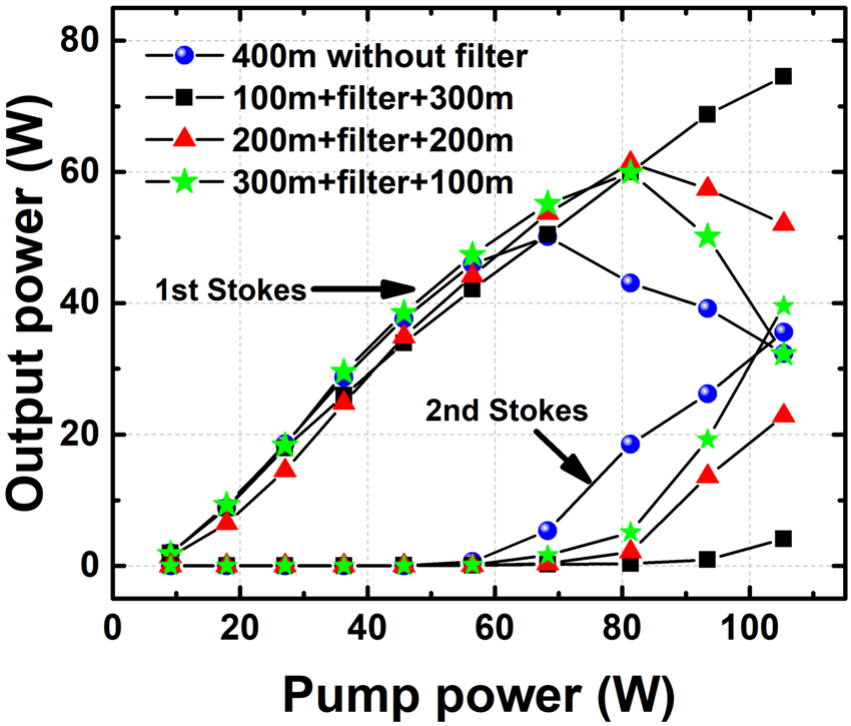
The output power curve of the first order and second order Stokes light at different positions.

Based on the results of proof-of-concept experiment above, an experiment on suppressing the second order Stokes was conducted. The output power curve of the first order and the second order Stokes light in Raman fiber amplifier with and without the filter at different positions is given in Fig. 5. The total output power was recorded by a power meter. The powers at the first and second Stokes were calculated by spectral integration. As the pump power increases, the output power of the first order Stokes increases at the beginning, and then decreases owing to the generation of second order Stokes. This means the second order Raman gain limits the power scaling of the first order Stokes light. The maximum output power of the first order Stokes light is 50.1 W without the filter, while the maximum power is increased by 48.7% to 74.5 W with the filter. As for the second order Stokes light, the maximum output power is 35.6 W in the absence of the filter, while it is decreased by 88.5% to 4.1 W. The output power with the filter at different positions were also compared and the length of 100 m was also the best in terms of suppression effect for PMF 1 in Fig. 3.

**Figure 6:**
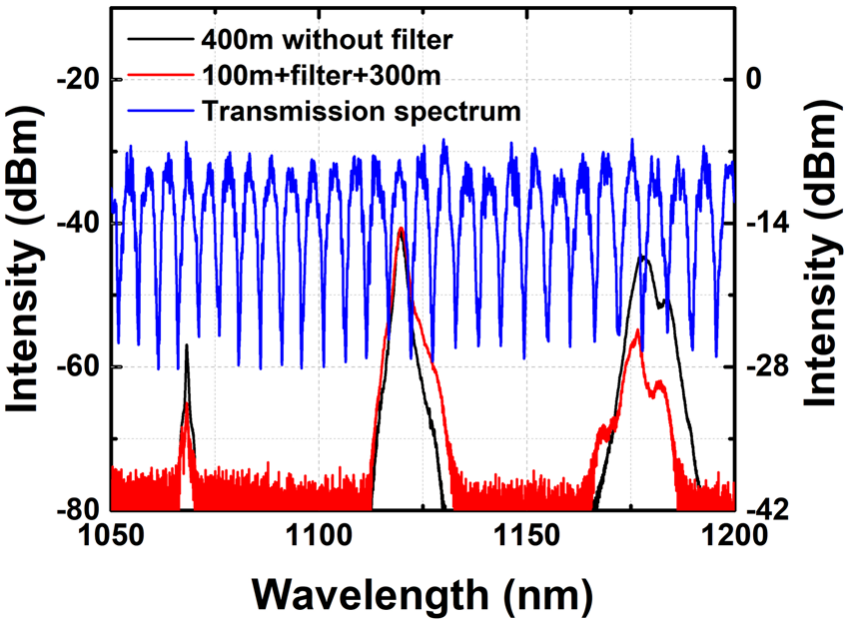
The spectrum in the experiment on suppressing the second order Stokes: The transmission spectrum of the filter, the output spectrum of the Raman laser at the maximum pump power without filter and that with filter when the length of PMF 1 is 100 m.

The spectral properties of the filter and the Raman fiber laser were both measured by the optical spectrum analyzer (OSA, Yokogawa AQ6370D). The spectrum of the above-mentioned ASE source could not cover the wavelength of the second order Stokes light (~1178 nm). Therefore, a home-made supercontinuum source was adopted to measure the filter’s characteristics. When the temperature is 20.1 °C, the transmission spectrum along the slow axis from 1050 nm to 1200 nm is shown in Fig. 6. By tailoring the fiber around 80 cm, the filter has high transmittance at pump light (1068.3 nm) and first order Stokes light (1119.6 nm) and low transmittance at second order Stokes light (1177.8 nm). Detailed transmission spectrum would be described later in this paper. This means that the polarization direction of both pump and the first order Stokes light would remain unchanged, while the polarization direction of the second order Stokes light would deflect by 90°. From the experimental results above, the suppression effect is the best at the length of 100 m for PMF 1. Under the maximum pump power, the output spectrum of the Raman fiber was measured and compared in Fig. 6. The intensity difference between the first and second order Stokes light is 3.5 dB, while that is up to 14.5 dB for the Raman fiber adopting a filter when the length of PMF 1 is 100 m. This difference also indicates that the filter has significant suppression effect. The two peaks in the output spectrum of second order Stokes light in the Raman fiber laser without a filter is consistent with the double-peak structure of Raman gain profile^14^. In terms of the spectrum of the Raman fiber laser with a filter, the second order Stokes light has three peaks, which corresponds to the wavelengths with high transmittance filter characteristics.

## Discussion and Conclusion

In contrast to other optical devices used to suppress Raman gain^19,22,24,26^, the spectral filter in the experiment has very simple structure, quite low cost, and extremely low insertion loss. A piece of PM passive fiber is sufficient to fabricate the filter. The main theoretical basis is the polarization-dependence of Raman gain, thus no light is transferred to the cladding mode, which results in nearly no heat accumulation. Therefore, the spectral filter has the potential to handle high power. By using this filter, the second order Stokes light was suppressed efficiently. The parameters in the Raman amplifier and the location of filter could all be further optimized, thus further power scaling of the first order Stokes light could be achieved.

In conclusion, we proposed a novel all-fiberized spectral filter in order to efficiently suppress high order Stokes light and increase the output power of the first order Stokes light in a linearly polarized Raman fiber laser. Through this filter, the polarization direction of high order Stokes light could be manipulated to be orthogonal to that of pump light. High order Stokes light would be suppressed because Raman gain is quite small when the pump light and Stokes light are orthogonally polarized. It is the first time that the polarization-dependence property of Raman gain has been used to suppress high-order Stokes light, to the best of our knowledge. The bandwidth and central wavelength could be tuned flexibly through changing the length and temperature of the cross-spliced fiber. The filter was employed in a linearly polarized Raman fiber amplifier, and finally the maximum output power of the first order Stokes light is 74.5 W, which is increased by 48.7%. In the next step, the suppression effect could be optimized by changing the length and temperature of the filter. The suppression effect of lumped spectral filters could also be explored. The parameters such as fiber length in the Raman fiber amplifier could be optimized to increase the conversion efficiency. In addition, the output properties could be real-time controlled by changing the difference of refractive index, for example, based on piezoelectric ceramic.

## Methods

### Physical principle of the filter

**Figure 7:**
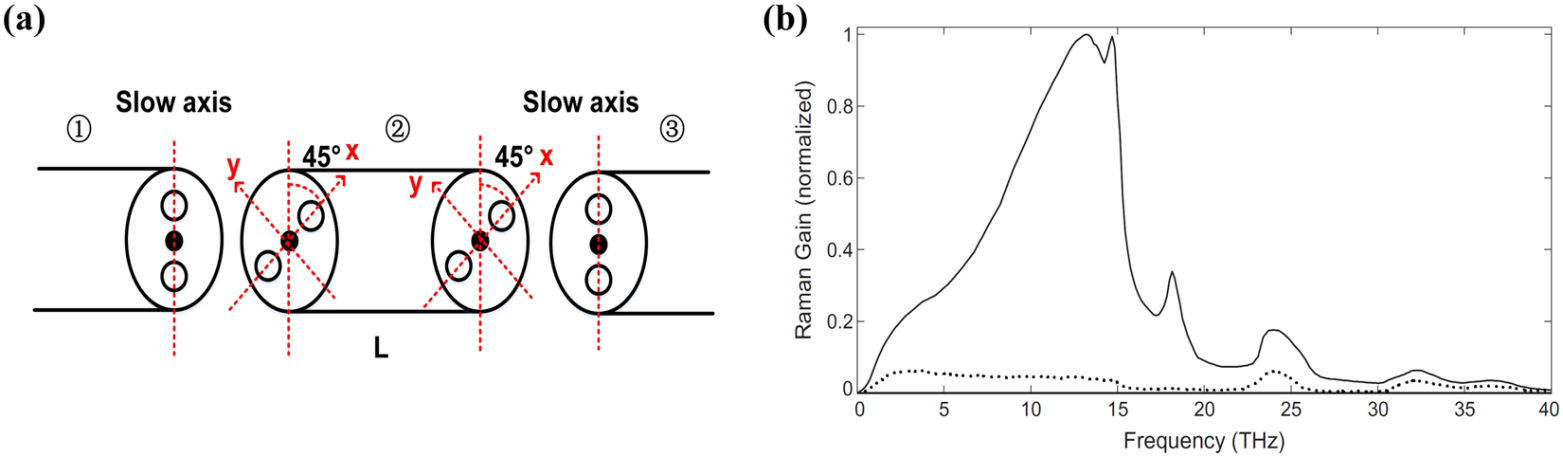
(**a**) The scheme of 45° cross-splicing of polarization maintaining fiber; (2) typical Raman gain spectrum for fused silica when the pump and Stokes light are copolarized (solid) and orthogonally polarized (dotted).

The scheme of the filter based on 45° cross-splicing of PANDA PM passive fiber is depicted in Fig. 7(a). The structure of this filter is similar to Lyot filter^27–29^, which is consisted of two polarizers and a piece of birefringent crystal with its principle axis at a 45 degree to the axes of both polarizers. Different from the Lyot filter, there is no polarizers in our filter, and the pump light is linearly polarized. Through the first splicing point, the incident light polarized in the slow axis direction would be decomposed into light in both directions along the fast and slow axes of fiber ②, where the difference of refractive index, Δ*n*, exists owing to birefringence. Then, after the transmission of fiber ②, the phase difference Δ*φ* between the light propagating in the fast and slow axes obeys the equation:

1 $${\rm{\Delta }}\phi =2\pi /\lambda {\rm{\Delta }}nL,$$

where $\lambda$ is wavelength, $L$ is the length of fiber ②. Another end of fiber ② is fused to fiber ③, and the difference of principle axis is also 45 degrees. When the phase difference ${\rm{\Delta }}{\rm{\phi }}=(2m+1)\pi$, where *m* is integer, the polarization direction of the light at the wavelength $\lambda$ would be rotated by 90° and the incident light would turn to propagate along the fast axis of fiber ③. When ${\rm{\Delta }}{\rm{\phi }}=2m\pi$, the polarization direction at the wavelength $\lambda$ would remain unchanged. In this case, this filter could be regarded as an all-fiberized flexible waveplate. According to the typical Raman gain profile in Fig. 7(b)^14^, Raman gain is very small when the pump and Stokes light are orthogonally polarized. Combination of the spectral filter and the polarization-dependence of Raman gain can be utilized to suppress specific order Stokes light.

The theoretical transmission spectrum and its influencing factors were also explored. Taking the slow axis of fiber ② as the x-axis in Fig. 7, the output laser after the filter can be expressed using Jones matrix as:

2 $${\rm{A}}=[\begin{array}{cc}{\cos }^{2}\theta  & \frac{1}{2}sin2\theta \\ \frac{1}{2}sin2\theta  & {\sin }^{2}\theta \end{array}]\,[\begin{array}{cc}{e}^{-i{\rm{\Delta }}\phi } & 0\\ 0 & 1\end{array}]\,[\begin{array}{c}\frac{\sqrt{2}}{2}\\ \frac{\sqrt{2}}{2}\end{array}],$$

where the matrices from right to left represent pump light polarized at a 45° degree to x-axis, PM fiber, and the projection on the slow axis of fiber ③ (*θ* is the angle between the slow axis of fiber ③ and x-axis, namely 45°). Then the transmittance is calculated to be

3 $$T=\frac{1}{2}+\frac{1}{2}cos({\rm{\Delta }}\phi ).$$

Two adjacent peaks in the transmission spectrum satisfy the formula:

4 $$m{\lambda }_{m}={\rm{\Delta }}nL,$$

5 $$(m+1){\lambda }_{m+1}={\rm{\Delta }}nL$$

where $m$ is integer. Then the bandwidth of the filter is

6 $${\rm{\Delta }}\lambda \approx \frac{{\lambda }^{2}}{{\rm{\Delta }}nL}.$$

**Figure 8:**
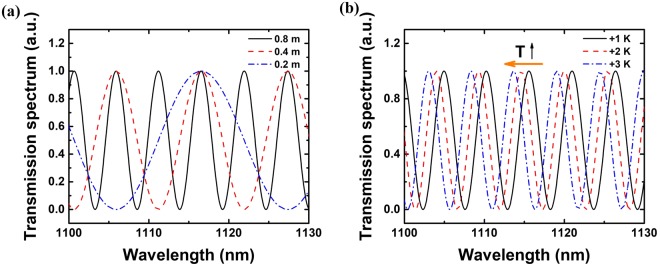
The theoretical transmission spectrum versus (**a**) length and (**b**) temperature.

Therefore, the bandwidth of this filter could be tuned by changing the length of the cross-spliced fiber. As is shown in Fig. 8(a), as the length of fiber ② decreases, the bandwidth of the filter becomes broader and the interval between adjacent peaks becomes larger. The phase difference Δ*φ* is proportional to wavelength $\lambda$ and the difference of refractive index Δ*n* (~0.0003). At the same time, the difference of refractive index ${\rm{\Delta }}n$ is temperature dependent and the temperature sensitivity of modal birefringence $d({\rm{\Delta }}n)/dT$ is ~−10^−8^/K^30,31^. The theoretical scheme of the transmission spectrum of the filter as a function of temperature T is shown in Fig. 8(b). As the temperature T increases, the transmission spectrum shows blue-shift trend. By controlling the length and temperature of fiber ②, the wavelength selective characteristics of the filter could be tuned. Therefore, the filter that would make the polarization direction of high order Stokes light orthogonal to that of pump light could be demonstrated after specific design.

### Measurement method of transmission properties of the filter

A home-made ASE source and a home-made supercontinuum source were employed to provide broad band pumping in the proof-of-concept experiment and experiment on suppressing the second order Stokes light, respectively. The pump source was polarized by a PBS, the polarization extinction ratio of which is around 20 dB. The slow-axis port of the PBS was selected to be spliced to the spectral filter because the polarization direction of the pump source in the Raman amplifier is along the slow axis. Another port is angle-cleaved by 8° to avoid unwanted feedback and thus protect the pump source. The length of the cross-spliced 10/125 μm (core and cladding diameters) PM passive fiber was designed to be 80 cm, and the bandwidth of the spectral filter was calculated to be around 0.5 nm. The polarization direction of light at some wavelengths would deviate from the slow axis. Then a PBS was adopted to extract the light propagating along the slow axis. The output end of the slow-axis port of this PBS is also angle-cleaved by 8° and the output spectrum is recorded by an OSA.

### Experimental method on suppression of the second order Stokes light

Owing to the principle of the spectral filter, it is better to make the light pass unidirectionally. Therefore, the filter was inserted into a Raman fiber amplifier. The pump laser is a hundred-watt level tunable linearly polarized Yb-doped fiber amplifier with MOPA configuration, the seed of which has an optical tunable filter and a polarizer. The polarization direction of slow axis was chosen by the polarizer and the polarization extinction ratio of the pump laser is around 20 dB. The pump laser has 25 nm tuning range from 1055 to 1080 nm. The tuning property of pump source was specially designed to make it easier to select the length of the cross-spliced fiber in the filter. In this case, attention only need to be paid on the transmittance of first and second order Stokes light, while that of the pump light could be adjusted by tuning the pump wavelength. A home-made linearly polarized Yb-doped fiber laser centering at 1119.6 nm was used as the seed. LP lasing was obtained through polarization-dependent bending loss^32^, and the polarization extinction ratio was around 19 dB. The pump laser and the seed were connected to the 1070-nm port and 1120-nm port of the 1070/1120 nm WDM, respectively. A piece of commercial PM passive fiber was spliced to the common port of WDM. And the spectral filter was spliced after it. Another piece of PM passive fiber was connected after the filter to make the whole length a constant for easy comparison. The output end of the Raman amplifier is angle-cleaved by 8° in order to avoid unwanted feedback.
